# Assignment of the disordered, proline-rich N-terminal domain of the tumour suppressor p53 protein using ^1^H^N^ and ^1^H^α^-detected NMR measurements

**DOI:** 10.1007/s12104-023-10160-4

**Published:** 2023-10-20

**Authors:** Fanni Sebák, Péter Ecsédi, László Nyitray, Andrea Bodor

**Affiliations:** 1 Analytical and BioNMR Laboratory, Institute of Chemistry, Eötvös Loránd University, Pázmány Péter sétány 1/a, Budapest, 1117 Hungary; 2 Department of Biochemistry, Eötvös Loránd University, Pázmány Péter sétány 1/c, Budapest, 1117 Hungary

**Keywords:** Intrinsically disordered proteins, p53, Proline-rich domain, ^1^H^α^-detection, Real-time homo- and heteronuclear decoupling acquisition

## Abstract

Protein p53 is mostly known for playing a key role in tumour suppression, and mutations in the p53 gene are amongst the most frequent genomic events accompanying oncogenic transformation. Continuous research is conducted to target disordered proteins/protein regions for cancer therapy, for which atomic level information is also necessary. The disordered N-terminal part of p53 contains the transactivation and the proline-rich domains—which besides being abundant in proline residues—contains repetitive Pro-Ala motifs. NMR assignment of such repetitive, proline-rich regions is challenging due to the lack of amide protons in the ^1^H^N^-detected approaches, as well as due to the small chemical shift dispersion. In the present study we perform the full assignment of the p53^1–100^ region by applying a combination of ^1^H^N^- and ^1^H^α^-detected NMR experiments. We also show the increased information content when using real-time homo- and heteronuclear decoupled acquisition schemes. On the other hand, we highlight the presence of minor proline species, and using Pro-selective experiments we determine the corresponding *cis* or *trans* conformation. Secondary chemical shifts for (C^α^–C^β^) atoms indicate the disordered nature of this region, with expected helical tendency for the TAD1 region. As the role of the proline-rich domain is yet not well understood our results can contribute to further successful investigations.

## Biological context

The tumour suppressor p53 protein (human p53 UniProt ID: P04637) is a well-known transcriptional factor regulating key cellular processes upon genotoxic stress: controls DNA damage repair mechanisms, triggers cell cycle arrest and induces apoptosis in the nucleus or in the cytosol (Green and Kroemer 2009; Follis et al. 2015). The loss of p53 function results in uncontrolled cell growth. In normal cells, p53 level is low, and it is regulated by murine double minute 2 (MDM2) ubiquitin ligase. This interaction is a major cancer therapy target (Espadinha et al. 2022; Koo et al. 2022; Vassilev et al. 2004).

**Fig. 1:**
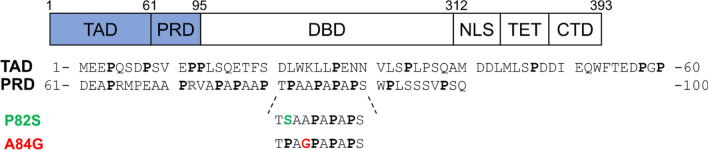
Domain structure of p53 protein (Krois et al. 2016) with the sequence of p53^1–100^. The studied mutations, P82S and A84G are highlighted with green and red colours. Note, that the P72R natural polymorph was used for all p53 variants in this study. *TAD* transactivation domain, *PRD* proline-rich domain, *DBD* DNA-binding domain, *NLS* nuclear localization signal, *TET* tetramerization domain, *CTD* C-terminal regulatory domain

In its functional state, the 393 residue-long p53 is a homotetramer. Each monomer contains all main functional domains (Fig. 1). The N-terminal transactivation domain (TAD) is highly mobile and disordered with nascent helical elements (Wells et al. 2008). This region is followed by the proline-rich domain (PRD). Previous studies suggest that PRD plays mainly a structural role (Wells et al. 2008; Toledo et al. 2007), however several hot spot mutations are localized between residues 55–100 (72, 73, 82, 84, 89, 98) (Hoyos et al. 2022). Residues involved in the mutations can be found in PXXP sequence repeats (X: any amino acids) which is the consensusSH3 domain interaction site. The mutations can alter several protein-protein interactions (Toledo et al. 2006; Berger et al. 2001). On the other hand, it has been revealed that *cis-trans* isomerization of Pro82 by Pin1 is essential for consequent phosphorylation on Ser20 by checkpoint kinase 2 (CHK-2). Thus, p53 sensitivity to inhibition by MDM-2 increases (Berger et al. 2005).

The N-terminal TAD and PRD region of p53 protein has been investigated by NMR with various sequence length and sample conditions, however, full-assignment of the proline-rich region has not been achieved (BMRB ID: 17760, 50960, 51124) (Wong et al. 2009; Usluer et al. 2021; Mandal et al. 2022). All studies showed successful assignment for more than 95% of the TAD domain (1–60), but proline resonance assignment and the full assignment of the repetitive motifs in PRD region is missing.

Here, we aim to further explore the N-terminal region of p53^1–100^ towards better understanding the interactions of the proline-rich region. We report the first, full, solution state backbone assignment of the wild type p53^1–100^ and minor conformers arising from proline *cis-trans* isomerization using recently published ^1^H^N^ and ^1^H^α^-detected NMR approaches combined with real-time homo- and heteronuclear decoupling schemes.

^1^H^N^-detection does not to provide full assignment of disordered proteins, especially if the sequence is abundant in proline residues—which lack the amide proton. A solution to this is ^1^H^α^ -detection, where all residues are detectable. Another favourable feature of this approach is that H^α^ is a not an exchangeable proton, therefore it is suitable for samples at physiological pH and temperature (Mantylahti et al. 2010; Wong et al. 2020; Bodor et al. 2020). In the present study, we used the previously introduced sensitive, high resolution 2D SHACA-HSQC including the BASEREX hetero- and homonuclear decoupling scheme during acquisition (Haller et al. 2019; Bodor et al. 2020). BASEREX was also applied in various 3D experiments to improve resolution (Sebák et al. 2022; Szabó et al. 2022). Determination of the exact proline conformation was done by the selective Pro-(H)CBCGCAHA experiment. On the other hand, in case of ^1^H^N^-detection we applied the BEST-TROSY measurement incorporating real-time pure shift acquisition containing the ^13^C-BIRD^X^ inversion element to increase spectral resolution in peak picking (Haller et al. 2022).

## Methods and experiments

### Protein expression and purification

Human p53^1–100^ (UniProt code: P04637) was cloned into a modified pGEX vector (pETARA) containing an N-terminal TEV-cleavable glutathione S-transferase (GST) tag. This construct was modified using the Quickchange mutagenesis technique to generate the p53^1–100(P82S)^ and p53^1–100(A84G)^ point mutants.

The different ^13^C/^15^ N-labelled p53 peptides were expressed and purified as previously (Dudás et al. 2020; Sebák et al. 2022). The constructs were transformed into *Escherichia coli* BL21(DE) cells and the cultures were grown in Luria-Bertani (LB) broth complemented with 100 µg/mL ampicillin. Before induction the cells were transferred into a minimal broth containing 50 mM Na_2_HPO_4_, 20 mM KH_2_PO_4_, 8.5 mM NaCl, 1 mM CaCl_2_, 2 mM MgSO_4_, 18.7 mM NH_4_Cl and 22.2 mM glucose (in ^15^N/^13^C-labelled form). The expression was induced with 0.25 mM isopropyl β-d-1-thiogalactopyranoside (IPTG) and the cells were incubated at 28 °C for 3 h. Pelleted cells were disintegrated by ultrasonication in a buffer containing 20 mM Tris, pH 8, 300 mM NaCl, 0.1 mM tris(2-carboxyethyl)phosphine (TCEP). Cell lysates were clarified by centrifugation at 48,000×*g*. The supernatants were loaded onto Protino Glutathione Agarose 4B resin (Macherey-Nagel) and the p53 constructs were eluted using 10 mM reduced glutathione in the buffer. The GST tag was eliminated using TEV protease at room temperature, overnight. After complete cleavage, GST was removed from solution by heat denaturation followed by centrifugation. P53 fragments were further purified by reversed-phase HPLC on a Jupiter 300 C5 column (Phenomenex). The peptide containing fractions were lyophilized and stored at − 80 °C. All peptides contain additional 2 residues on the N-terminus (Gly-Ser) from cloning, the assignment of these residues is not included in the study.

### NMR spectroscopy

NMR samples of p53^1–100^ variants (wt, A84G, P82S) contained 1 mM ^13^C, ^15^N-labelled protein, 20 mM MES, 20 mM NaCl, 10 mM TCEP, 3 mM NaN_3_, 10% D_2_O, 1% DSS at pH 6.0. All NMR spectra were recorded on a Bruker Avance III 700 spectrometer operating at 700.05 MHz using a Prodigy TCI H&F-C/N-D, z-gradient probe-head. ^1^H chemical shifts were referenced to the internal DSS standard, whereas ^15^N and ^13^C chemical shifts were referenced indirectly via the gyromagnetic ratios. Temperature was calibrated against the methanol standard sample (Findeisen et al. 2007). All measurements were performed at 298 K.

Peak assignment and sequential connectivities were determined from ^1^H^N^- and ^1^H^α^-detected experiments: 2D ^1^H,^15^ N-BEST-HSQC and ^1^H,^15^ N-BEST-TROSY (Haller et al. 2022) and 3D BEST-type HNCACB, HN(CO)CACB and HNCO (Lescop et al. 2007). High resolution, ^1^H^α^ -detected 2D SHACA-HSQC spectra (Bodor et al. 2020) were recorded for proline peak detection, 3D HCAN and HCACON spectra were used for unambiguous peak assignment for ^1^H^α^-^13^C^α^ correlation-based measurements (Kanelis et al. 2000; Szabó et al. 2022). Proline conformation was determined from 3D Pro-(H)CBCGCAHA measurements (Sebák et al. 2022). Experimental details and acquisition parameters are provided in Table 1. All spectra were processed with TopSpin 3.6.2 and analysed with CARA 1.8.4.2.(Keller 2004).

**Table 1:** NMR experimental parameters enabling resonance assignment of p53^1–100^ and P82S, A84G mutants

Experiment	Dimension of acquired data given as real points			Spectral width/ppm			Recycle delay/s	Number of scans
	F3	F2	F1	F3	F2	F1		
2D ^1^H,^15^N-BEST-HSQC	–	2048	512	–	12	24	0.2	2
2D ^1^H,^15^N-BEST-TROSY*	–	4096	1024	–	12	24	0.2	8
3D BEST-HNCACB	2048	80	128	12	30	80	0,2	8
3D BEST-HN(CO)CACB	1024	80	64	12	30	80	0.2	24
3D BEST-HNCO	2048	64	80	12	30	20	0.2	8
2D SHACA-HSQC*	–	4096	1024	–	12	20	0.7	2
3D HCAN*	4096	104	64	12	25	35	1	8
3D HCACON	2048	80	64	12	30	35	1	8
3D Pro- (H)CBCGCAHA*	4096	512	16	12	18	18	1	4

∗With real-time homo- and heteronuclear decoupling acquisition scheme

## Extent of assignments and data deposition

**Fig. 2:**
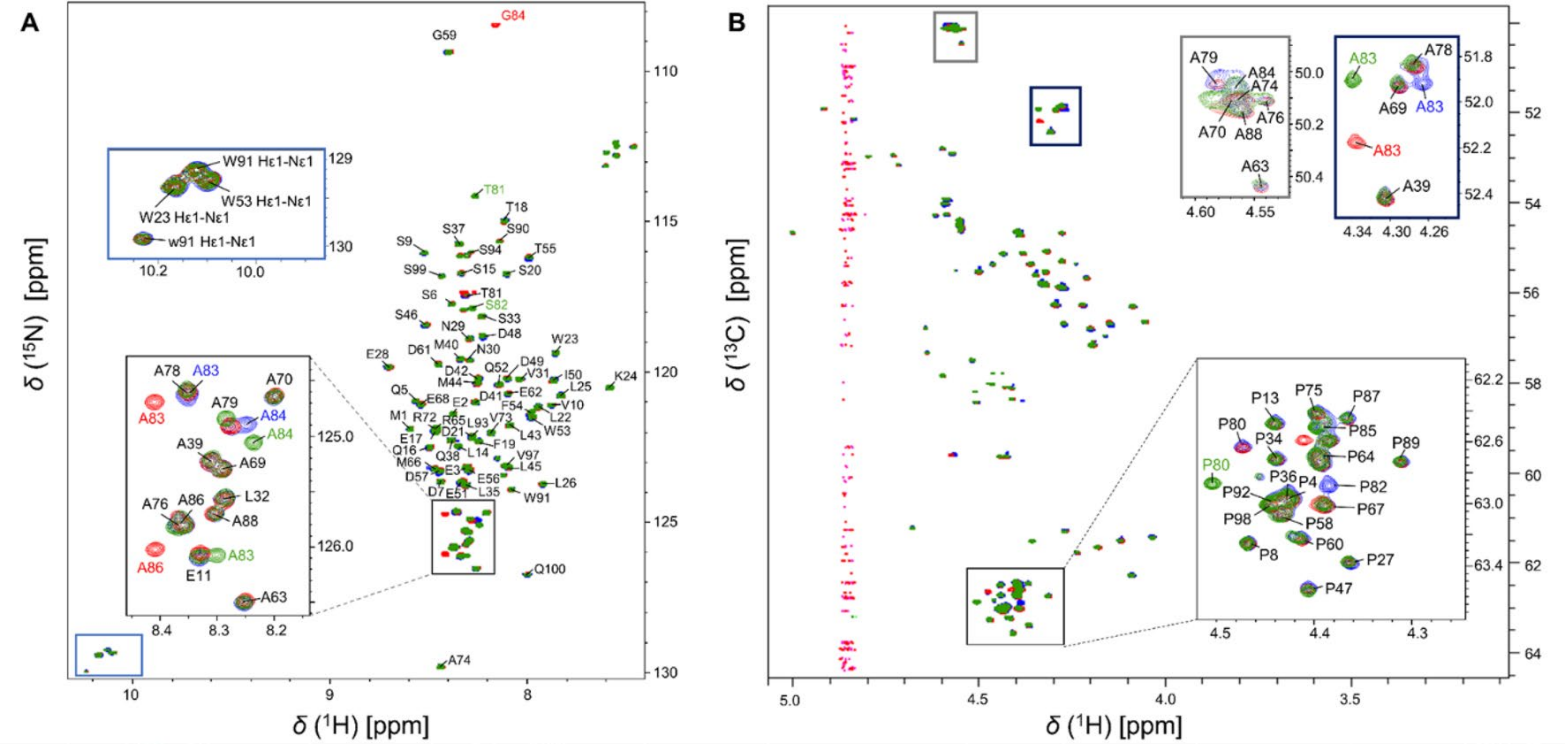
2D ^1^H,^15^N-BEST-HSQC (**A**) and SHACA-HSQC (**B**) spectra of the p53^1–100^ variants: wild type (blue), A84G (red) and P82S (green). Zoomed insets show the crowded Ala and Pro-rich parts of the BEST-HSQC and SHACA-HSQC spectra, as well as the Trp sidechain H^ε1^-N^ε1^ signals. The minor peaks are labelled with lowercase letters

p53^1–100^ protein is intrinsically disordered with repetitive Ala and Pro-rich motifs; thus, the signal dispersion is narrow in the ^1^H,^15^N-HSQC spectrum causing severe signal overlap (Fig. 2). The real-time homo- and heteronuclear decoupling acquisition schemes help in obtaining an increased spectral resolution (see experiments in Table 1). This allowed us to separate peaks even in the Ala-rich region. Still, this was still not sufficient for unambiguous resonance assignment. In this respect two mutations—both bearing biological relevance—were chosen: A84G and P82S. The introduction of these mutations cause perturbation in the chemical environment of the neighbouring peaks on the ^1^H,^15^N-HSQC spectra, and this helps to perform a 97% assignment of backbone ^1^H^N^ and ^15^N resonances of the non-proline residues (76 of 78) (Fig. 2A). Only the peaks of Ser 95 and Ser 96 could not be unambiguously distinguished.

Proline residues were characterized using the ^1^H^α^-detected approach. The high resolution 2D SHACA-HSQC spectrum allows to distinguish the 22 proline residues (Fig. 2B). Sequential connectivities were done using 3D HCAN and HCACON spectra. ^1^H^α^-detected 2 and 3D measurements allowed 90% assignment of all residues (90 of 100). The 10 unassigned residues involve Gly59 which is not detected on the 2D SHACA-HSQC, and several distorted serine residues (94–96) still overlap. Resonance overlap hinders unambiguous assignment of 2 prolines and 1 alanine in the PRD region, as well as 2 Asp and 1 Glu residues. The type of proline isomer was determined from the C^β^ and C^γ^ chemical shift difference, being ~ 5 ppm for *trans*, and ~ 10 ppm for the *cis*-Pro isomer (Schubert et al. 2002). For this purpose, the 3D Pro- (H)CBCGCAHA spectrum was recorded which correlates the proline sidechain C^β^ and C^γ^ signals with the H^α^–C^α^ crosspeaks. Results indicate that the major proline peaks are exclusively *trans* isomers.

**Fig. 3:**
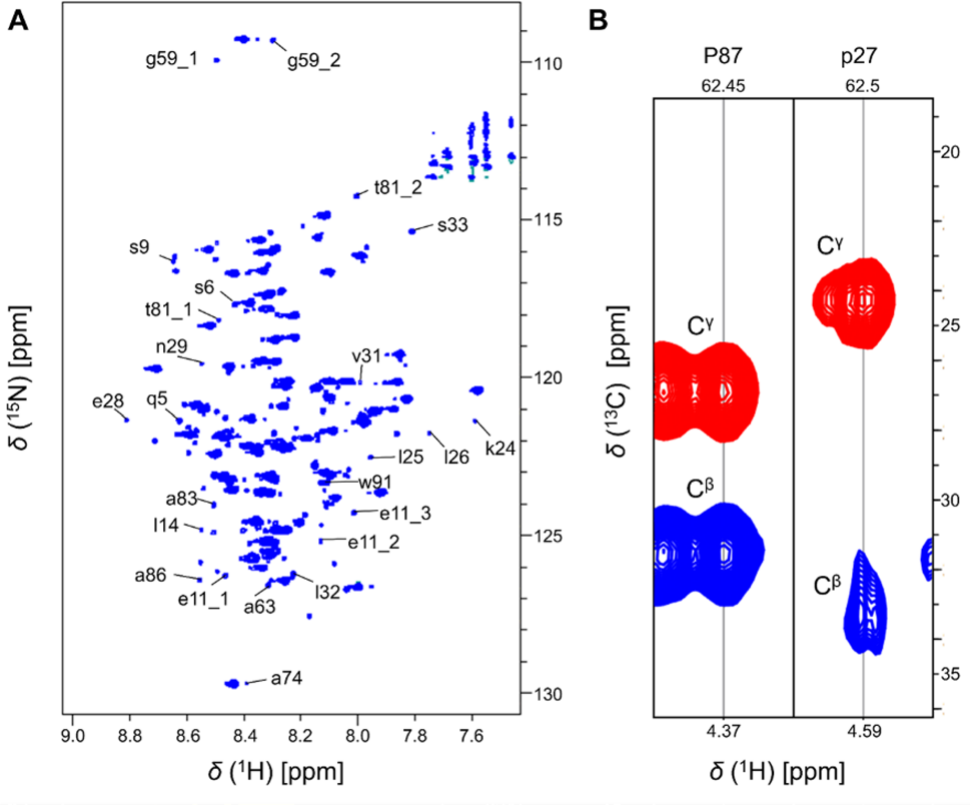
Minor peaks arising from proline *cis-trans* isomerization: zoomed ^1^H,^15^N-BEST-TROSY spectrum of the p53^1–100^ at 298 K (**A**) and example strips for *trans* and *cis*-Pro from the 3D Pro(H)CBCGCAHA spectrum (**B**). Minor peaks are marked with lowercase letters

As the disordered p53^1–100^ is highly enriched in prolines (22 out of 100 residues), several small intensity minor peaks arise from the Pro *cis-trans* isomerization (Fig. 3). The high resolution 2D BEST-TROSY spectrum allowed the detection of more than 40 minor peaks, however due to the signal overlap in the 3D spectra and the repetitive sequence, the assignment of only 24 peaks was successful. In our previous work, the assignment of the minor peaks in p53^1–60^ was published (Sebák et al. 2022), these peaks also appear for the p53^1–100^ variant. As the amount of these minor conformers is between 3 and 15%, we can conclude that ratio of these minor peaks is not affected by the longer protein sequence.

In the PRD region the assignment was successful for several minor peaks. Proline isomerisation results in the appearance of minor peaks also for the neighbouring residues. For example, in the case of Pro80-Thr81-Pro82 segment - besides the major peaks - two minor peaks were detected for Thr81. The major peaks are assigned, as expected as the *trans-*Pro80-Thr81-*trans*-Pro82 segment. For the minor peaks the following connectivites are revealed: *cis*-pro80_1-thr81_1 fragment, as *δ*(^13^C^β^) ~ 34.2 ppm for pro80_1 is detected, and the succeeding proline is most probably *trans*-pro82, though the detection of this peak was not possible. Similarly, the other minor fragment is *trans*-pro80_2-thr81_2, as *δ*(^13^C^β^) ~ 31.7 ppm for pro80_2 is observed. The existence of thr81_2 is a consequence of the proline 82 *cis*-*trans* isomerization. This observation is strengthened by the disappearance of this minor peak for the P82S mutant. Regarding the amount of the two minor peaks: these are 3.2% and 7.0% respectively. The polar Thr81 residue in the Pro preceding position slightly increases the *cis-*Pro82 amount (Sebák et al. 2022).

The most intensive minor peak belongs to Trp91, as in this case the interaction between the aromatic sidechain and the Pro92 is energetically favourable. Consequently, the amount of the minor peak is more than 30%, which is in agreement with previous literature findings (Sebák et al. 2023). Most minor peak intensities in the repetitive Pro- and Ala-rich region are ~ 3–10% of the corresponding major conformer, however, unambiguous assignment is not possible. In the A84G mutant, again two minor peaks arise for Gly84 with intensities 11.7% and 7.7%, respectively.

In conclusion, using the combination of ^1^H-detected approaches, 100% assignment of C^α^ resonances was possible, including both Pro and non-Pro residues. Moreover, characterization of minor species is also given.

**Fig. 4:**
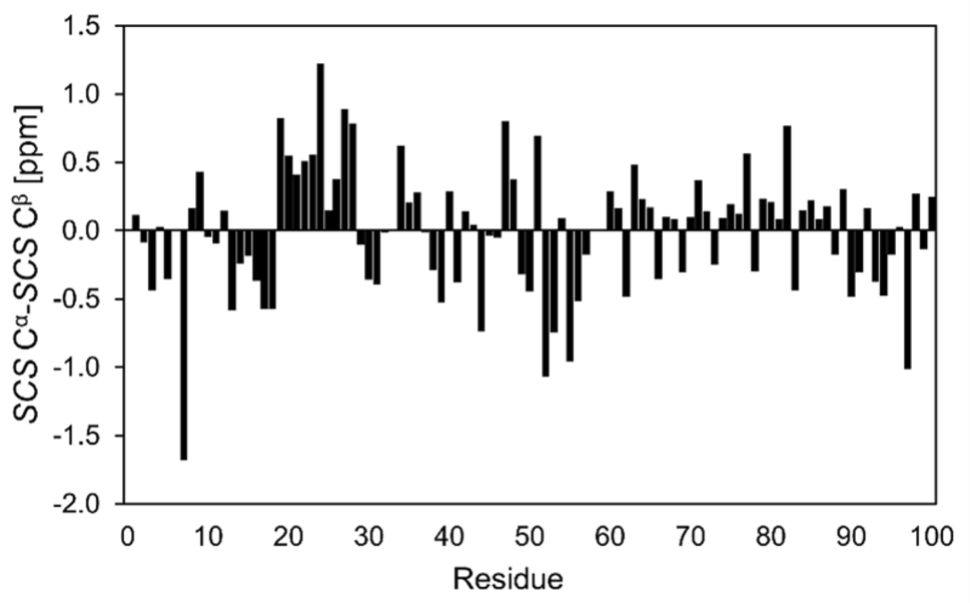
Secondary chemical shifts analysis reveals that the major conformer of p53^1–100^ is highly disordered throughout the entire protein sequence, transient helical tendency can be observed only in the Ser20-Pro27 region

Further on, the obtained C^α^ and C^β^ chemical shifts were used for secondary chemical shift calculations (*SCS*) using the following equation: *SCS* = *δ*_measured _− *δ*_random coil_. Random coil chemical shifts and neighbour corrections were derived from Kjaergaard et al. (Kjaergaard et al. 2011; Kjaergaard and Poulsen 2011). The calculated chemical shifts predicts the p53^1–100^ protein to be highly disordered with a nascent helicity in TAD1 region (Ser20- Pro27)(Fig. 4.), in accordance with earlier findings.

## Data Availability

The ^1^H, ^13^C and ^15^N backbone and sidechain chemical shifts of p53^1–100^ have been deposited in the Biological Magnetic Resonance Data Bank (BMRB) under the accession number 51984.

## Abbreviations

BASEREX: Band-selective refocusing on the X-nuclei
CHK-2: Checkpoint kinase 2
PRD: Proline rich domain
TAD: Transactivation domain
